# Effect of Negative Pressure Therapy on Open Abdomen Treatments. Prospective Randomized Study With Two Commercial Negative Pressure Systems

**DOI:** 10.3389/fsurg.2020.596056

**Published:** 2021-02-05

**Authors:** Thomas Auer, Siegfried Sauseng, Pavle Delcev, Peter Kohek

**Affiliations:** General, Visceral and Transplant Surgery, Department of Surgery, Medical University of Graz, Graz, Austria

**Keywords:** open abdomen therapy, abdomen vac therapy, abdomen sepsis, abdominal compartment syndrome, negative pressure on bowel surface, Suprasorb CNP_R_, ABthera_R_

## Abstract

**Introduction:** The use of negative pressure dressings for open abdominal therapy has made a great impact on strategies for open abdominal treatment. Observed intestinal damage and developement of fistula formation raises questions about safety of commonly used systems (AB-Thera). The most common used system uses foils for shielding intestines directly from negative pressure. As an alternative a system with open pore dressing in double layer film was introduced (Suprasorb CNP) and proved to safe in animal studies. We compared the effects of this two systems on patients requiring open abdominal treatment.

**Materials and methods:** Patients with secondary peritonitis in at least two abdominal quadrants were included in this randomized study. Inclusion criteria were secondary peritonitis (ACS), abdominal compartment syndrome, and abdominal trauma combined with ACS and/or contaminated abdomen. Patients with active bleeding and pancreatitis were not included. We examined Mannheim peritonitis Index (MPI), bloodcount, PCT, amount of fluid collected, and morphological changes on the bowel. Data were collected on day 2, 4, 7, 14, 21, and 28. Primary end point was fascial closure. Examination was terminated in case of death and damage to the abdominal organs. Groups were compared using Mann Whitney *U-*test and chi square test. Trend evaluation was evaluated using an one way repeated measure analysis of variance. *P-*values below 0.05 was considered significat.

**Results:** Thirty four patients were included between August 2010 and September 2012. There were no significant difference between two groups in MPI, age, and gender. Mean duration of treatment, WBC, CRP, and abdominal closure rate were not significantly different between groups. Suprasorb CNP System collected twice more fluid than AB-Thera and decreased PCT on significantly faster rate than AB-Thera. Four patients died (11%) and four patients developed enteric fistula (11%). Closure rate was achieved in 27 out of 34 Patients (79.5%). Closure rate was not significantly different between groups.

**Conclusion:** The use of both systems proved to be efficient and safe. The application of well-dosed, moderate negative pressure on contaminated areas of the abdomen seems to have a lot of potential and it is worth directing greater research potential in this direction.

## Introduction

**Figure 1:**
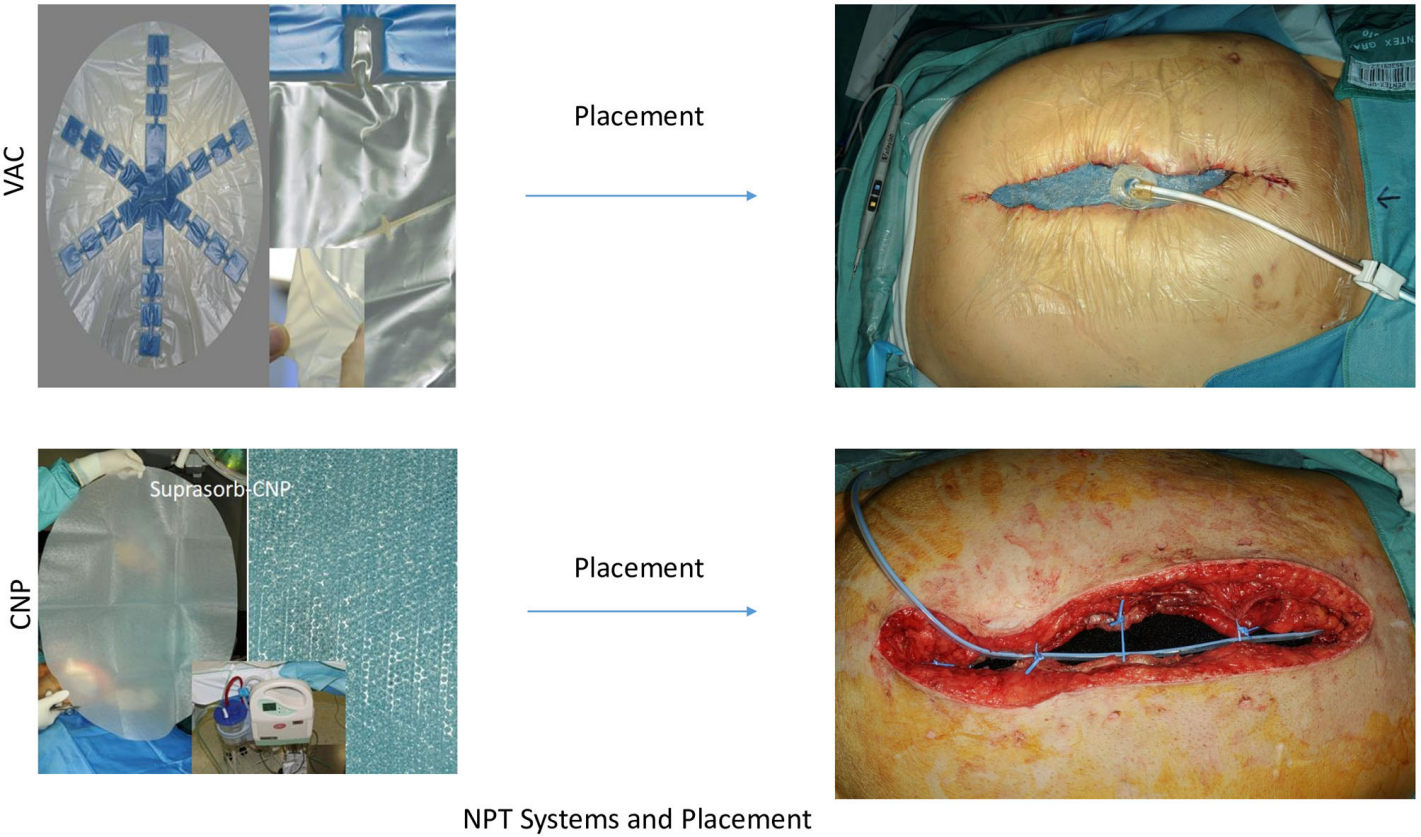
AB-Thera^®^ NP-Dressing, Suprasorb-CNP^®^ Dressing.

The use of negative pressure dressings for open abdominal therapy (OAT) was probably first described by Brock 1995 (1) and has influenced the development of strategies for treatment of secondary peritonitis (SP) and abdominal compartment syndrome (ACS). Without any doubt, negative pressure therapy (NPT) systems offer a new dimension in OAT, fulfilling most of the criteria for optimizing success and minimizing risks in OAT (2). Nevertheless, the controversy between open abdomen treatment and “en demand” strategy with the risk of tertiary peritonitis is inherent in the therapy strategies. Opponents of OAT can also rightly point out that there are no guidelines for an exact indication and technical processes. Additionally reports of intestinal damage, fistula formation, can cause uncertainty about the use of OAT treatments with NPT (3–7). The question arises whether the currently widespread systems actually represent the only and correct philosophy or if there is still potential in the further development of the NPT systems. The most widespread system, AB-Thera^®^ (ABThera system, KCI, San Antonio, Texas, USA) (Figure 1), and most commercial applications, use soft foils to protect the intestinal bundle, and only sparse openings to keep the negative pressure away from the intestinal surfaces (8). Opposite to these systems, we use a second film system, Suprasorb-CNP^®^ (Suprasorb CNP system, Lohmann & Rauscher, Austria-Germany) (Figure 1), which protects the intestinal surfaces through soft material properties, but remains permeable to the negative pressure. In a preclinical animal study we have examined this system to determine whether the effect of negative pressure on the surface of the intestine and on organs causes damage (9). This system works with closely spaced pores in a double-layer film. In our *in vitro* study, this system showed the double drainage effect to the AB-Thera film (10).

In this study, the effects of both systems are compared. In addition, the sum of both systems should show how effectively NPT therapy works in a controlled study conducted by surgeons with special experience in open abdomen treatment, on patients of different degrees of severity.

## Materials and Methods

Thirty-four patients were included during 2.5 years in an “Intention to treat” protocol. Patients were randomly assigned to experimental groups using the web-based randomizer (11). The inclusion was carried out without any influence from the treating surgeon by calling up the selection decision on the web-based randomizer. The study followed the rules approved by the ethics committee of the Medical University of Graz, Austria (No.: 21-198, 08/09).

The AB-Thera^®^ system, referred to as VAC-system, consisted of polyurethane-foam (PUF) in star form, welded onto a fenestrated plastic film (Figure 1). This was inserted in the abdomen covering the greater omentum and the whole intestine up to the liver and down into the pelvic cavity. The 1.5 cm pre-shaped PUF-oval was placed over this protecting contact layer and positioned 3–4 cm below the edges of the inner abdominal wall. There were 3–4 vessel loops^®^ (Vessel loops, Devan, Covidien, USA) used as single stitches to approximate the muscle-fascia layers as a kind of dynamic retention suture (12). The subcutaneous space was filled with a second layer of 1.5 cm pre-shaped PUF-oval, attached to the skin's edges with staples. The wound was closed with the system's adhesive drape. Using a fixed suction line and suction pump, a negative pressure of −125 mmHg was maintained in all cases in accordance with the company's recommendations (Figure 1).

The Suprasorb-CNP^®^ system, referred to as S-CNP-system (Figure 1) used a membrane as described above, shielding the intestine, liver surface and pelvic cavity (Supplementary Material, L&R product description). The film was covered with 1.5 cm PUF and 3–4 dynamic sutures were placed exactly as forementioned in the VAC system. In this system, however, a perforated silicon drainage tube was placed in this plane and connected with the suction pump, served as the suction line (Figure 1). After filling the subcutaneous space with Kerlix^®^-gauze (Kerlix-Gauze, Covidien, USA), the skin around the wound was covered with a few layers of Kerlix^®^-gauze and then closed with the adhesive drape. A negative pressure of −60 mmHg (−50 to −80 mmHg) was maintained, according to the cited reference animal study (9).

### Inclusion Criteria

**Figure 2:**
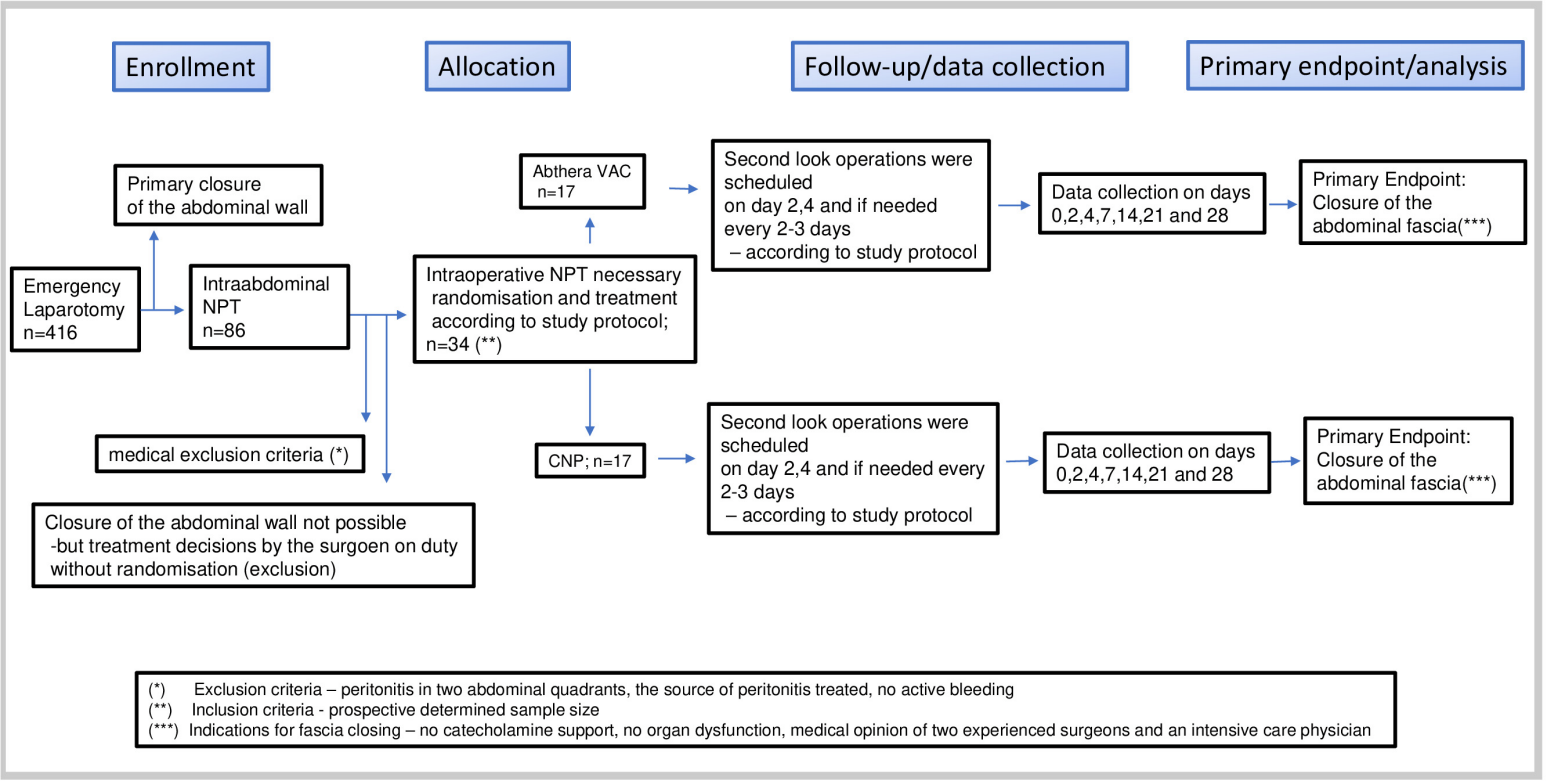
Study flow diagram according to CONSORT NPT.

Flow diagram (Figure 2).

– Patients with secondary peritonitis in at least two abdominal quadrants were included, when the cause of the peritonitis (source) had been found and treated. The decision for open abdominal treatment was made by the surgeon on duty. Criteria for the decision were defined as follows:
– Patients who had exhibited peritonitis for more than 24 h and in whom a second look was planned or for whom the abdomen could not be closed for other reasons;
– Patients presenting with ACS for whom the indication for open abdominal treatment after failure of conservative treatment was made, when they had been otherwise stabilized and no active bleeding was present;
– Patients after abdominal trauma combined with ACS and/or contaminated abdomen due to enteral perforation, when they had been stabilized, and no active bleeding was present.

### Exclusion Criteria

– Patients with pancreatitis as the source of peritonitis
– Patients with active abdominal bleeding
– Pregnancy
– Patients under 18 years of age.

Whenever a patient developed an obvious entero-atmospheric fistula, the observation was terminated and subsequent treatment was given outside this study. If enteric opening was observed we repaired it with sutures as usual, and gave a “c” according to the amended OA classification (13), as described below in the secondary parameters. If the opening persisted after the 2nd attempt at repair, it was then categorized as fistula and marked with “4,” according to same classification (13). Those patients were then excluded from further observations in this study.

### Study Design

The Mannheim Peritonitis Index (MPI) (14–18) was determined for every patient at the time of inclusion. During and after the operation, photographic records were made on the following objects:

– OP-site before and after treating the source of peritonitis
– The development during NPT and the condition of dressings after application and before removal.

Changes of dressings with abdominal lavage were planned in the operating room on days 2, 4, and every 2–3 days thereafter.

Data collections were performed on days 0, 2, 4, 7, 14, 21, and 28 in Examinations 1–7 (E1–E7).

### Primary End Point

Primary end point was defined as closure of the muscle-fascia- abdominal wall before or on day 28. The examination of all patients for this study was terminated on day 28. The follow-up regarding the death was continued for the entire inpatient process.

### Secondary Examination Parameters

1. Age, gender and BMI distribution for both groups.
2. MPI at the time of inclusion of the patient. According to published data of predicted mortality and MPI values, a cut-off point was set to a value of 25 MPI points to show the distribution of low and high risk patients of both groups (14, 17). To facilitate the comparison of the distribution, MPI classification was divided into 4 groups according to the severity of peritonitis and concomitant parameters.
3. Medical history and diagnosis relevant for inclusion: E1
4. Blood cell count and chemistry: Leucocytes, C-reactive protein (CRP), Pro-calcitonin (PCT), at every examination.
5. Amount of fluids collected per 24 h via the NPT System: E2–end.
6. Damage to the abdominal organs and tissue caused by the NPT system: E2–end.
7. Open abdomen classification (13): E1–End (Abdominal closure, premature termination).

Criteria for abdominal closure:

– Patients' clinical state had to improve to the extent that they were free of catecholamine support and no longer had any major organ dysfunction requiring external support (ventilation, hemofiltration).
– The inflammatory parameters tend to normalize.
– Two experienced surgeons with the involvement of the responsible intensive care physician decided whether the abdomen was ready for closure.

### Statistical Analysis

We did a pilot study including 17 patients per group based on the following sample size considerations. A sample size of 17 in each group will have 80% power to detect a difference in means of 7 (the difference between a Group 1 mean, μ1, of 14 and a Group 2 mean, μ_2_, of 7) assuming that the common standard deviation is 7 using a two group *t-*test with a 5% two-sided significance level.

The data obtained for patients were mean, median, standard deviation (stand.dev.), minimum (min), and maximum (max) for continuous variables and absolute and relative frequency for categorical data. The differences between the two groups were analyzed using the Mann-Whitney *U-*test and the chi-square test as appropriate. To compare trends in the inflammation parameters, a one-way repeated measure analysis of variance was used. We performed a linear mixed model analysis for the rank-transformed PCT values using patient as random effect and group (S-CNP or VAC) as well as a linear trend over time as fixed effects. A *p-*value below 0.05 was considered significant. The software package SPSS 20.0.0 was used for statistical analysis.

## Results

Thirty-four patients were included, 17 in each group.

Overall there were 22 male and 12 female patients with a median age of 59.5 years (range: 23–79).

The distribution of age, gender, MPI, MPI range, and BMI for both groups is shown in Table 1.

**Table 1:** A: Age, B: Gender, C: MPI, D: MPI range, and E: BMI distribution for both groups.

	**A: Age**			**B: Gender**		**C: MPI**			**D: MPI**	**Range**		**E: BMI**		
	**Mean**	**Min/Max**	**Stand.dev**.	**Male**	**Female**	**Mean**	**Min/Max**	**Stand.dev**.	**0–25**	**25–30**	**>30**	**Mean**	**Min/Max**	**Stand.dev**.
VAC	57.1	23/76	17.4	12	5	25	12/36	8.1	7	5	5	31	196/484	7.66
		Sign.:	*p =* 0.45	Sign.:	*p =* 0.721		Sign.:	0.241					Sign.:	0.031
S-CNP	52.8	23/79	15.4	10	7	29	12/43	9	7	3	7	25	176/355	4.335

The causes of peritonitis and indications for open abdominal treatment are listed in Table 2. Lower intestine defects were more frequent in the S-CNP group (8 compared to 5) whereas upper intestine defects were equally frequent in both groups.

**Table 2:** Diagnoses and sources of peritonitis.

**Diagnoses**	**VAC**	**S-CNP**	**n-total**
Abdominal trauma with rupture and/or necrosis in the colo-rectal area, traumatic gastric perforation		3	3
Spontaneous and post-operative liver abscess	2		2
Perforated appendicitis with peritonitis	4	1	5
Perforation, anastomotic rupture in the colon, sigmoid colon, and rectum.	5	8	13
Gastro- duodenal ulcer perforation	2	1	3
Small bowel perforation, anastomosis rupture, uro-conduit necrosis.	3	4	7
Abdominal compartment syndrome	1		1
***n***	17	17	34

The MPI values showed in Table 1C. were only slightly different, the difference was not statistically significant.

The distributions of MPI values below and above 25 (Table 1D) were equal for both groups. Values higher than 30 occurred more often in the S-CNP group (7 vs. 5, respectively). The difference was not significant.

The values of BMI are displayed in Table 1E. The difference between the groups was significant. Two severely obese patients were found with a BMI of 48 in the VAC group, while an underweight patient with a BMI of only 17 was found in the S-CNP group. The BMI was involved to observe the influence on fistula formation and mortality (Tables 3, 4).

**Table 3:** Patients who diet during study observation or hospital stay after study termination.

	**Pt.No./Age**	**MPI**	**BMI**	**Days E1 to closure or termination**	**Days E1 to +**	**Diagnosis comments**
	5/50	36	19.6	1	1	Liver abscess, Leucemia, Sepsis, MOF
**VAC**	10/68	32	24.2	4	173	Duodenal fistula, Parkinson‘s disease
	17/77	30	33.1	15	43	Bladder cancer, Gangrene of the small bowel
**S-CNP**	29/79	37	27.5	10	13	Late treatment of tubo-ovarian abscess, MOF

*Comparative parameters (Age, MPI, BMI). Days from therapy onset to termination and to death, commented main, and accompanying diagnoses*.

**Table 4:** Patients developing enteric fistulae.

**Nr**	**Study duration- d**	**MPI**	**BMI**	**Location of fistula**	**NP-system**
1	12	12	34.2	Small bowel	VAC
10	4	32	24.2	Duodenum	VAC
12	5	34	48.4	Ileo-transversostomy	VAC
14	5	29	33.1	Small bowel	VAC

*Days from therapy onset to termination due to fistula formation, MPI- levels, BMI, and location of fistula formation. All observations were in the VAC group*.

The mean duration of treatment (Table 5A) was found to be 6.6 days with VAC and 8.9 days with S-CNP. Although the maximum treatment duration was longer for S-CNP than VAC (25 and 15 days, respectively). The difference between the two groups was not significant.

**Table 5:** A: Duration of treatments (E1—closure or termination), B: Fluid samples collected per 24 h.

	**A: Duration of treatments/days**			**B: Total fluid volume/ml**		
	**Mean**	**Min/max**	**Stand.dev**.	**Mean**	**Min/max**	**Stand.dev**.
VAC	6.6	1/15	3.7	1981.3	220/6,900	1669.7
		Sign.: 0.532			Sign.: 0.004	
S-CNP	8.9	2/25	6.9	3779.4	850/10,700	2250.1

Fluid collections during 24 h before examinations are shown in Table 5B. With the S-CNP treatment, about twice the amount of fluids was delivered than with the VAC system. The difference was statistically significant (*p* = 0.004).

All descriptive statistics for Leucocytes and CRP can be found summarized in the Supplementary Table 1.

The values of leukocytes and CRP showed a continuous downward trend in both systems. There were also increases in both groups. At one measuring point, E3, a significantly lower value could be recorded for CRP in the VAC system, but this was not confirmed at the following measuring points. Overall, no specifically useful course could be found for leukocytes and CRP.

Descriptive statistics for PCT values are summarized in the addended Table 2.

In the PCT values, both groups showed a linear decrease in the values at the successive measuring points. This showed a significance of <0.001 for both. The differences in the values between the groups were clear, the PCT values for VAC were significantly higher at all measuring points than those for S-CNP, *p* = 0.034.

**Figure 3:**
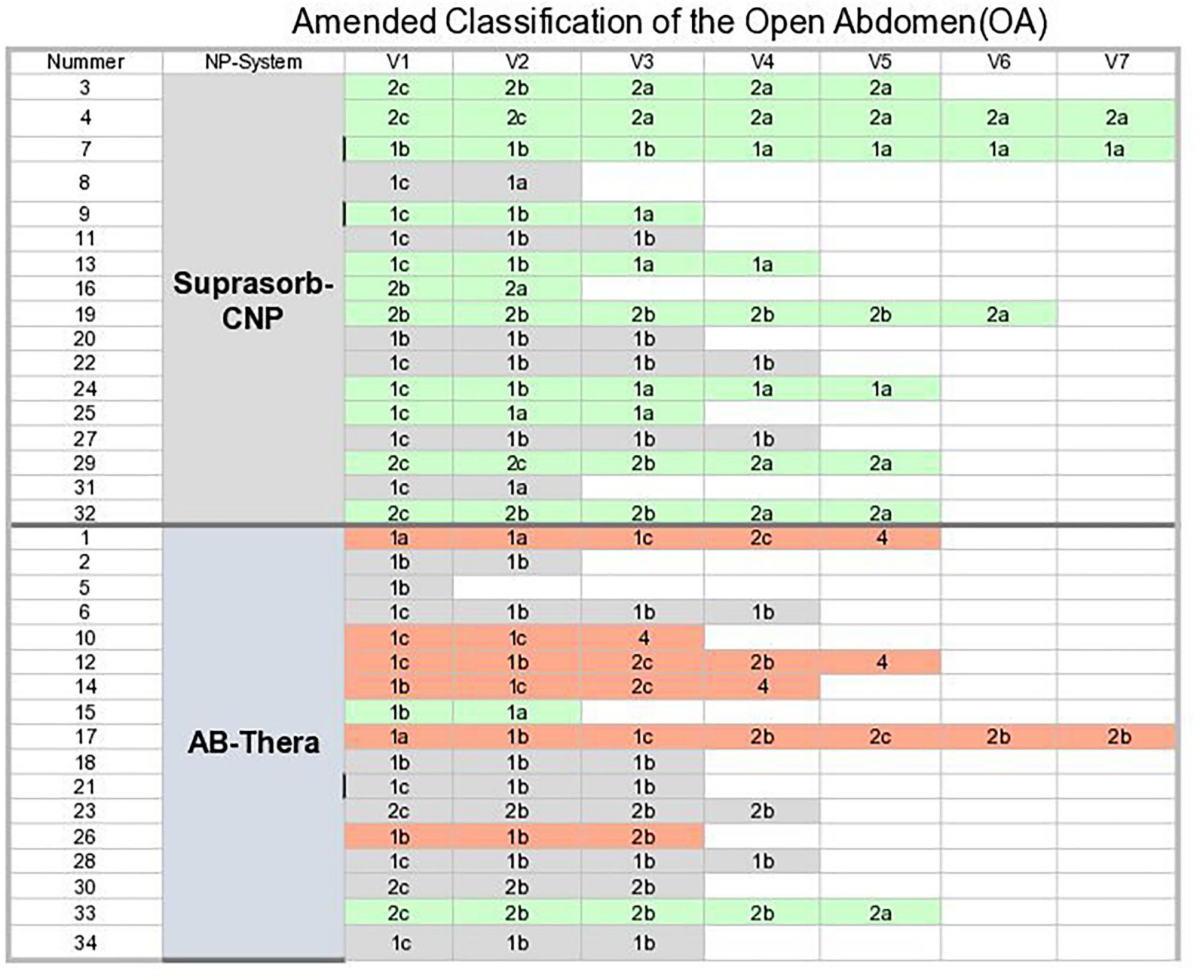
Summary of the “Amended open abdomen classification.” Green, decreasing OAC grades; Gray, constant OAC grades; Orange, increasing OAC-grades.

A summary of the “Amended open abdomen classifications” (13) (OAC grades) of all patients at E1–End is shown in Figure 3. The dominant green for S-CNP indicates the tendency for decreasing OAC-grades, the dominant gray and red for CNP the tendency for constant and increasing OAC-grades.

The difference of tendencies of OA grades for both system groups in **Table 7**. was found to be significant.

Early termination of study treatments:

Four patients were excluded from further study participation when they developed enteric fistulae. Details are listed in Table 4.

A total of 4 patients in this study died (Table 3). One patient, a 59 years old female, died on the 1st post-operative day of fulminant sepsis due to liver abscess in a myeloid leukemia disease with the appearance of acute multi-organ failure. Two patients died after abdominal consolidation and a closed abdominal wall in the combination of their multiple morbidity and the additional burden of their septic abdominal disease. One patient died on the 173rd post-operative day after initial sewing of a duodenal perforation. The study observation had to be ended on the 4th day after the 2nd NPT dressing change because of fistula formation of the over-sewing. The subsequent treatment outside of the study showed no success and the patient very slowly developed a multi-organ failure. All 4 patients were found with MPI >30.

The overall mortality rate was found to be 11.76% (4 out of 34), 1 before and 3 after abdominal wall closure, 3 in VAC group, 1 in the S-CNP group. All of them were part of the MPI >29 group therefore the mortality rate in this specific group was 26.6%.

The primary end point, the closure of the muscle-fascial abdominal wall (**Table 8**), was achieved in 27 out of 34 patients (79.54%), after a mean of 7 days of treatment. Treatments ended with definitive closure of the abdominal wall in 70.6% of the VAC group and 88.2% (n.s.) of the S-CNP group. In 2 patients, due to trauma-related necrosis of the rectus muscles, the fascia could only be closed by bridging with prosthetic material. Both of them were in the S-CNP group and they were not included into the abdominal wall closed group.

There was no significant relationship between MPI, days of treatment and abdominal closure.

## Discussion

In this study, 33 of the consecutive patients were included with secondary peritonitis and 1 patient with abdominal compartment (Table 2). Hence, this can be viewed as a peritonitis study. The severity according to the MPI was slightly higher in the S-CNP group (n.s.), but in both groups it was clearly in the range of higher severity, MPI median 28 and 29 (Tables 1C,D). The mortality in this range of MPI grades is indicated as about 44% (17). The selective mortality in this group MPI >29 in this study is 26.6% and thus a clear signal for the benefits of NPT treatment in this indication group. In the MPI <25 group, 17% mortality is listed (17) while in the present study this group shows no mortality. The distribution between the systems for this MPI grades is equal, 7(VAC) and 7 (S-CNP) (Table 1).

The results are of course also to be assessed with regard to the performance of intensive care medicine and its progress since 1994.

Inflammation parameters are known to have an accompanying significance as a decision-making aid in the treatment of septic patients. Three common parameters used in the routine of intensive treatment: white blood cell count, CRP, and PCT were tested for their usefulness in NPT. PCT has been described as the most accurate and specific parameter (19–23). Our study confirmed PCT as the best predictive parameter.

The PCT values of both systems showed a significant linear decline, a fairly clear vote for the use of NPT in septic abdomen. However, the difference between the two systems was very clear here: the PCT values of S-CNP were significantly lower overall than with the VAC system (Table 6, Supplementary Table 2). The interpretation of the possible importance is discussed later in the overview.

**Table 6:** Mixed model analysis for the rank-transformed PCT values using patient as random effect and group (S-CNP or VAC) as well as a linear trend over time as fixed effects.

	**Mean rank (95% CI)**	***p-*value**
Intercept	71.1 (57.3, 84.8)	<0.001
VAC/S-CNP group	20.0 (2.36, 37.7)	0.034
Visit	−10.8 (−13.3, −8.3)	<0.001

The data for the other two inflammation parameters, leukocytes and CRP, were of no use for a specific follow-up of the course of the disease under NPT. No knowledge could be gained by comparing the two systems.

Fluid management, a fundamental requirement of OAT (24), can be described as uncomplicated in both systems and as satisfactory from a patient care point of view. However, the evacuated amount of fluids was significantly higher with S-CNP than with VAC (Table 5B), practically to the same extent as was observed in an *in vitro* study (10). Since the rapid evacuation of infectious material is one of the basic requirements for septic abdominal treatment (25, 26), this can be seen as a clear advantage between the two systems.

To objectify and describe the condition of septic abdomen treatment, it is necessary to translate visual perceptions into comparable data. Even if the assessment was carried out by 2 surgeons on the basis of photos presented, the study could not be blinded. This must surely be seen as a weak point in the methodology. In this study, the “amended” score system by Björck et al. (13) was used for classification. The better clarification between “septic abdomen” and enteric leakage in the amended version of the OA classification compared with the original version (13, 27) on one side, there leaves still an area open where an enteric opening to a fistula manifests. A solution for this study was found by setting the definition of a fistula after two unsuccessful attempts at closure.

Figure 3 shows the results after the OAC grading, illustrated by a colored background. In the percentage representation (Table 7) the proportion of descending OAC grades is lower for the VAC group than in the S-CNP group; the difference is statistically significant. The proportion of constant OAC grades is higher in the VAC group than in the s-CNP group. The high proportion of ascending OAC grades in the VAC group is mainly due to the fact, that all 4 fistulas that occured were in the VAC group (Table 4). Apart from this, together with the significantly higher evacuated amounts of liquid and the observation of significant lower PCT values, the careful conclusion can be drawn that a reduced amount of negative pressure on the contaminated surfaces, including the intestinal surfaces, can be of therapeutic benefit compared to the shielding.

**Table 7:** Percentage of constant, increasing and decreasing amended open abdomen classification grades for both groups.

	**Decreasing %**	**Constant %**	**Increasing %**
VAC	25	44	31
	*p =* 0.008		
S-CNP	71	29	0

Even if all fistula formations are recorded in the VAC group, the chance factor cannot be ruled out given the small number of cases. An additional factor could also be 3 out of 4 overweight patients in this group, with 1 patient having a BMI of 48 (Table 4). Conversely, this study does not support the often anticipated fear that negative pressure on the intestinal surface is the reason for fistula formation (6). The total fistula rate of 11.7% is in the good normal range for abdominal sepsis, 5–20% as learned from the literature (3, 6, 7, 28).

The total abdominal wall closure rate of almost 80% (Table 8) is a very high value when measured against rates without the use of an NPT system of 12–24% (29, 30). The average closure rate with NPT systems was found about 70% (3–5, 7). The factors of the consistent additional use of a dynamic fascia anti-retraction system (12) and the work of a continuously competent team still seem to have this potential for improvement. The comparison of the closure rates of both systems of 70.6 (VAC) and 88.2 (S-CNP) is not significant. In both patients in the S-CNP group, where no primary closure could take place, the reason was the necrosis of the rectus muscles due to the underlying abdominal trauma and the abdominal wall could only be closed by bridging with the help of mesh prosthesis. The speculative assumption of these two patients as the primary closure would lead to an occlusion rate of 100% in this group. This should be considered especially under the aspect that in this S-CNP group only a negative pressure of maximum −80 mmHg was used. The negative pressure does not seem to play a major role as an anti-retraction factor and there is still potential for conventional strategies in this area.

**Table 8:** Muscle facia closure rate, statistics.

	***n***	**Days E1—closure Mean, min/max**	**%**
**VAC**	12	6.6, 2/15	70.6
		*p =* 0.396	
**S-CNP**	15	7.5, 2/25	88.2

*Closure rate combined (VAC+S-CNP) was 79.4%*.

The results of this study provide potential evidence that NPT may be useful in OAT. Due to the low number of cases, the data cannot expect any definitive statements. However, the partly significant results indicate that the negative pressure in the abdomen does not end when the wound of the abdominal cavity is treated while the intestine is protected from noteworthy negative pressure effects. The application of well-dosed, moderate negative pressure on contaminated areas of the abdomen shows a lot of potential and it is worth of further research.

## Data Availability Statement

The original contributions presented in the study are included in the article/Supplementary Material, further inquiries can be directed to the corresponding author/s.

## Ethics Statement

The studies involving human participants were reviewed and approved by ethics committee of the Medical University of Graz, Austria (No.: 21-198, 08/09). The patients/participants provided their written informed consent to participate in this study. Written informed consent was obtained from the individual(s) for the publication of any potentially identifiable images or data included in this article.

## Author's Note

The study showed that the negative pressure applied to the surface of the intestine has a stronger effect in infected areas than shielding them from the negative pressure. It was important for the author to determine whether this led to the widespread myth of accumulated fistula formation. Since this has not been observed, he would like to understand this as a step away from this myth and urge all interested open abdomen researchers to take a step in this new direction.

## Author Contributions

All authors listed have made a substantial, direct and intellectual contribution to the work, and approved it for publication.

## Conflict of Interest

The authors declare that the research was conducted in the absence of any commercial or financial relationships that could be construed as a potential conflict of interest.
