# Supplementary material for: Effect of Negative Pressure Therapy on Open Abdomen Treatments. Prospective Randomized Study With Two Commercial Negative Pressure Systems
Source: Front Surg. 2021 Feb 5;7:596056. doi: 10.3389/fsurg.2020.596056 (PMC7894571; doi:10.3389/fsurg.2020.596056)
Supplement: Supplementary file 1 [file Table_1.docx]

Supplementary Table 1.: Descriptive statistics for Leucocytes and CRP values of Examinations (E).
